# Relationships between psychological characteristics, academic fit and engagement with academic performance in veterinary medical students

**DOI:** 10.1186/s12917-023-03695-0

**Published:** 2023-08-24

**Authors:** Edlira Muca, Monica Molino, Chiara Ghislieri, Mario Baratta, Rosangela Odore, Domenico Bergero, Emanuela Valle

**Affiliations:** 1 Department of Veterinary Sciences, University of Turin, Grugliasco, 10095 Italy; 2 Department of Psychology, University of Turin, Turin, 10124 Italy; 3 Department of Veterinary Sciences, University of Parma, Parma, 43100 Italy

**Keywords:** Psychological characteristics, Academic fit, Well-being, Academic performance, Veterinary students

## Abstract

**Background:**

Recognition of the factors that influence academic performance in university students constitutes one of the key objectives of education researchers. Few studies have been conducted in this sphere in relation to veterinary students; however, considering the high levels of depression, anxiety symptoms, and decreased life satisfaction revealed in recent literature for this demographic, understanding these factors is of great importance. Moreover, the literature on veterinary education has mostly focused on cognitive factors as antecedents to academic performance, while very little attention has been directed toward personal characteristics.

**Methods:**

The present cross-sectional study aims to investigate the relationships between psychological characteristics (internal locus of control and self-efficacy), academic fit, well-being (engagement and exhaustion), and academic performance (average grade) among veterinary students. The study was conducted in the Department of Veterinary Sciences at the University of Turin between September 2021 and January 2022 involving 231 students.

**Results:**

The results of the Structural Equation Model confirmed a positive relationship between both internal locus of control and self-efficacy and academic fit, which in turn showed a positive relationship with engagement and a negative relationship with exhaustion. Finally, a significant positive relationship between engagement and academic performance was highlighted. Indirect effects were also significant, confirming the mediating role of academic fit and engagement.

**Conclusions:**

The study contributes to the literature by demonstrating the direct and indirect relationships among the variables selected in a sample group of veterinary students. These findings provide information for practical interventions that could support the academic experience and prospects of veterinary students by improving their psychological parameters and well-being.

## Background

Identifying the factors that influence academic performance in university students constitutes one of the key objectives of education researchers [1, 2] and also one of the main challenges faced by veterinary faculties [3, 4]. Education researchers have mainly focused on identifying the role of academic fit, internal locus of control, self-efficacy, engagement, and exhaustion in students’ learning processes and academic performance [5–8]. However, most of these studies have been conducted in the fields of medical education, psychology, social sciences and economics education, and as the curricula of these disciplines differ greatly from those studied by veterinary students, their results cannot be generalized to the veterinary context. Indeed, the educational environment as well as personal factors are known to affect learning, thus veterinary students’ learning styles are likely to differ from those of students from other disciplines [9]. Students’ personal factors – namely locus of control, self-efficacy, academic fit, and the sense of well-being – might also vary across different disciplines and academic environments, but this aspect has been largely neglected in veterinary education literature. Instead, research has tended to focus on cognitive factors, such as previous academic performance [10–13], as the key predictor of student outcomes across veterinary schools [14, 15]. However, these cognitive factors cannot explain much of the variance in academic performance [2, 16]. Yet, a growing body of veterinary literature indicates that a remarkably high percentage of veterinary students experience stressful situations during their education, resulting in depression, anxiety symptoms and decreased life satisfaction [17–20]. Nonetheless, there has been very little attempt to observe how these personal factors influence academic performance in veterinary students. Indeed, our knowledge about the effect of personal factors on veterinary students’ academic performance in the educational and clinical environment is very limited. Therefore, it is crucial that research be undertaken to fill this gap in the literature. This would allow us to understand how veterinary students manage events, challenges and stressors in their academic environment. It would also reveal the psychological factors that enable veterinary students to manage their academic tasks successfully, derive a strong sense of satisfaction from their studies, and maintain perseverance and enthusiasm even when facing challenges. Such knowledge would permit universities to improve veterinary education, in turn, helping their students to strengthen their capacities for academic achievement.

### The effect of internal locus of control on academic performance

Locus of control is one of the most researched personality characteristics in educational psychology. Its concept was first developed by Julian Rotter in 1966 as a two-dimensional personality trait consisting of internal and external orientation [21]. Individuals with an internal locus of control believe that events in their lives result primarily from their own actions. Alternatively, those with a strong external locus of control tend to believe that events in their lives are the result of external factors and circumstances beyond their control [21, 22]. Students with a strong internal locus of control believe that their behavior is closely linked with their academic performance and that their success is the result of their own efforts and actions [6]. By contrast, students with an external locus of control tend to blame everyone else for their failure [6]. Studies outside of veterinary education have demonstrated locus of control to have a significant positive effect on student academic performance [6, 23–25].

### The influence of academic self-efficacy on academic performance

Self-efficacy as a concept was firstly described by Bandura in 1977 in his social cognitive theory. It refers to an individual’s belief toward his or her own abilities to complete a given task successfully and achieve a desired goal [26, 27]. Self-efficacy in the academic context can be defined as a student’s beliefs toward their capabilities to achieve academic success and to accomplish specific academic goals [2, 28]. Self-efficacy is an important motivational factor which permits learners to achieve their specific goals through increasing their efforts, endeavor and perseverance [28–30].

In general, students with high levels of self-efficacy beliefs attribute their failures to poor attempts rather than low ability [2, 30, 31]. Conversely, students with low levels of self-efficacy beliefs attribute failure to their low capabilities as well as poor attempt and effort [2, 29]. Therefore, self-efficacy construct is assumed to be one of the most important predictors of student academic success.

### The effect of academic fit on academic performance

Person–environment fit focuses on the interaction and connection between person characteristics and the environment. According to this theory, the person not only influences his own environment, but the environment also affects the person [32]. Academic fit was firstly described by Schmitt et al. (2008) and refers to the person–environment fit in the context of an educational environment [5]. More specifically, it can be defined as how a chosen course fits a student’s interests and needs. Many studies have shown academic fit to be linked with both student academic satisfaction and academic performance [5, 33, 34]. Some researchers have suggested that recognizing a student’s academic fit can be beneficial to their academic achievement [5, 34, 35].

### The effect of student’s engagement on academic performance

Student engagement is described as the amount of physical and psychological energy students spend to stay involved and motivated to learn [36]. Conceptualized by Schaufeli and colleagues in 2002, it entails three dimensions: vigor, dedication, and absorption [37]. Students are vigorous when working on academic tasks, when they make greater effort and more attempts, when they persist in the presence of challenges, and when they have high energy levels and a positive approach to learning [38, 39]. Dedicated students find meaning and purpose in their academic tasks; they enjoy the challenge and experience inspiration, enthusiasm, and pride [38, 39]. Finally, absorbed students are entirely concentrated on their academic tasks and feel that time passes very quickly [38, 39]. Studies on all educational levels have demonstrated engagement to be a robust predictor of students’ academic performance [8, 37, 40–42].

### Conceptual framework and hypothesis

The theory of person–environment fit (P–E) is a useful framework for studying academic environments. The literature has consistently reported person–environment fit to be related to important outcomes [43–45]. Schmitt in 2008 conceptualized this theory in the educational context, naming it academic fit [5]. As hypothesized by Schmitt, person–environment fit – i.e., academic fit – influences learner satisfaction, which in turn influences academic performance [5].

Generally speaking, learner satisfaction as an emotional variable is directly correlated with an individual’s personal factors such as internal locus of control and self-efficacy beliefs. Bandura (1997) stated that self-efficacy was a key predictor of course satisfaction in students [26].

In 1999, Ponto investigated the role of locus of control on students’ satisfaction and revealed that students with an internal locus of control perceived more satisfaction than others [46]. Within veterinary education, Varnhagen and Wright investigated the role of locus of control in a distance-education program and revealed that internally oriented students were more satisfied with their learning experience [47]. Indeed, the positive effects of self-efficacy and internal locus of control on students’ satisfaction are evidenced by a large body of literature [46–49], while few attempts have been made to study student well-being; in particular, relatively little is known about the mediational role of work engagement and exhaustion.

Students who perceive a greater fit with the learning environment experience a positive learning process that in turn enhances their engagement, once again resulting in higher academic achievement. Moreover, academic fit might adversely affect student exhaustion, which generally results from a heavy academic workload and long study hours. It would be expected that students whose interests do not match the learning environment would experience a more negative learning process, potentially leading to exhaustion. Therefore, it can be assumed that the effect of academic fit on students’ academic performance can be mediated via student engagement and exhaustion.

Several studies have shown that engagement has a positive effect on academic performance [50–52]. The more engaged a student is, the higher his/her academic achievement.

On the contrary, student exhaustion negatively affects academic achievement. Specifically, exhaustion refers to the feeling that one’s emotional resources are overwhelmed by the high study demands. The veterinary curriculum is generally difficult and it requires long study hours to avoid university drop out, and the study program itself is also long. It is plausible to think that exhausted veterinary students may feel overwhelmed by a heavy academic workload.

Although many studies have been conducted on the direct relationships between variables such as internal locus of control, self-efficacy, academic fit, engagement, exhaustion, and academic performance, few studies have focused on the indirect relationships among them in a structural model. Previous research has investigated the effect of the above-mentioned variables on each other separately, and to the best of our knowledge no similar study has been conducted in the field of veterinary education. Therefore, the present study was conducted to investigate the association of internal locus of control, self-efficacy, academic fit, engagement, and exhaustion with veterinary students’ academic performance, considering also mediational effects. Specifically, it attempted to determine how academic fit can mediate the relationship between internal locus of control and self-efficacy on the one hand, and student engagement, exhaustion and academic performance on the other hand.

**Fig. 1:**
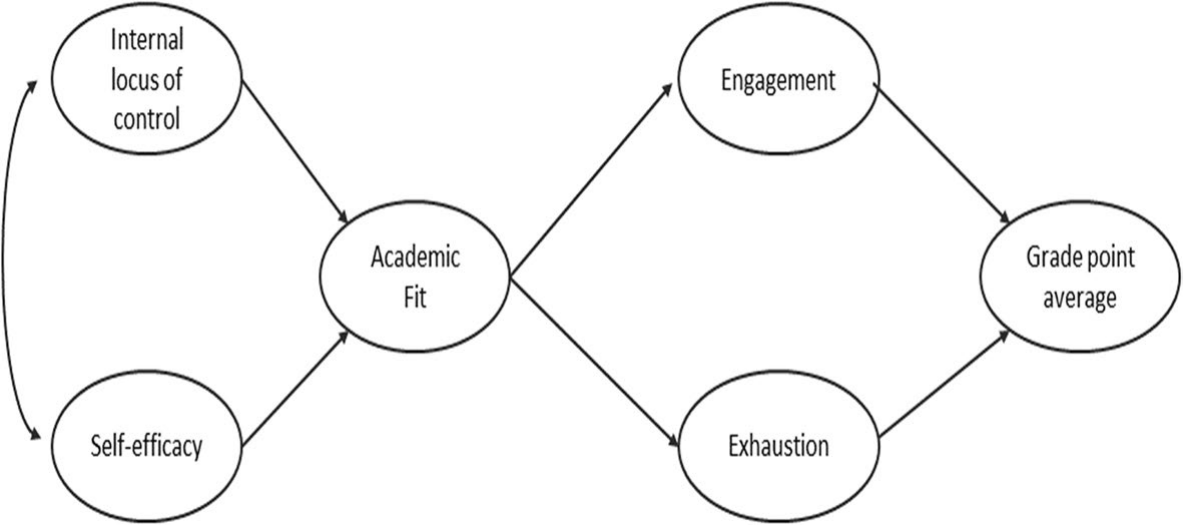
The conceptual model

The following research hypothesis and conceptual model are tested (see Fig. 1).

- Hypothesis 1: a) internal locus of control and b) self-efficacy are positively related to academic fit.
- Hypothesis 2: academic fit is a) positively related to engagement and b) negatively related to exhaustion.
- Hypothesis 3: a) engagement is positively related to academic performance and b) exhaustion is negatively related to academic performance.

## Material & methods

### Procedure and participants

This cross-sectional study was conducted in the Department of Veterinary Sciences at the University of Turin, Italy. The study’s participants were recruited from the first to the fourth years of the degree course in veterinary medicine (which involves 5 years of study; years 1 and 2 are dedicated to the basic sciences, years 3 and 4 to clinical sciences, and year 5 is the clinical trainship). They were asked to complete an online self-report questionnaire focused on specific psychological dimensions. Four hundred students received the questionnaire, which took approximately 10 to 15 min to complete and submit. Data was collected by distributing the questionnaire through a quick response code (QR) and web link using electronic platforms (Moodle, Facebook, WhatsApp). The questionnaire was uploaded onto the Uniquest platform, and the QR code and/or link was posted on the Facebook pages of the Department of Veterinary Medicine and the Moodle platform, as well as being distributed to students through WhatsApp groups. The first part of the questionnaire contained details about the study objective, the voluntary nature of participation, and the estimated time to complete the questionnaire. This was followed by the request for electronic consent, where students were given the option of completing the questionnaire or terminating the study. To agree to participation, they clicked on the “agree” button to indicate that they had read the above information and voluntarily agreed to participate; otherwise, they clicked on the “disagree” button to decline participation. Participant identity was not anonymous as the student serial number was requested in order to guarantee that only one profile from each student was submitted and to be able to offer the students individual feedback about their personal characteristics. Data was kept on the principal investigator’s personal computer using password protected files.

### Questionnaire development and data collection

A 17-item questionnaire was developed following an extensive review of the literature and with the collaboration of qualified psychologists. Their professional expertise allowed to identify and classify the most meaningful psychological characteristics and other elements indicated from the literature that may hinder veterinary students’ academic performance. The questionnaire was not pilot tested before use. The questionnaire was designed in the Italian language to address our intended aim of the study; all the scales used had already been validated in Italian or previously adapted for use in Italian studies.

Engagement was measured using the brief 9-item version of the Utrecht Work Engagement Scale for Students (UWES-S) [53], which applies a 7-point Likert-type scale (0 = never, 6 = every day). An example item is “When I’m studying, I feel mentally strong”. Cronbach’s alpha was 0.84.

Exhaustion was assessed using 5 items of the Maslach Burnout Inventory - Student Survey [53] which applies a 7-point Likert-type scale (0 = never, 6 = every day). An example item is “I feel emotionally drained by my studies”. Cronbach’s alpha was 0.86.

Academic fit was detected through 6 items [5] on a 5-point Likert-type scale (1 = very strongly disagree, 5 = very strongly agree). An example item is “I feel that my academic goals and needs are met by the faculty at this school”. Cronbach’s alpha was 0.79.

Internal locus of control was detected using 6 items [54] on a 5-point Likert-type scale (1 = very strongly disagree, 5 = very strongly agree). An example item is “There is a direct link between a person’s abilities and the position he/she holds”. Cronbach’s alpha was 0.73.

Self-efficacy was measured through 5 items [55] on a 5-point Likert-type scale (1 = very strongly disagree, 5 = very strongly agree). An example item is “I am confident that I will succeed”. Cronbach’s alpha was 0.80. Moreover, the questionnaire detected the GPA for each student by means of their serial number as a measure of their academic performance.

### Ethical considerations

Ethical approval was obtained from the University of Turin Research Ethics Committee [UOR: SI000045- Classif. III/11].

### Statistical analysis

Following data collection, the responses of the questionnaire were coded and inserted into a customized database using the Statistical Package for Social Sciences (SPSS 28). Descriptive data analysis, Pearson’s correlation and Cronbach’s alpha coefficients were calculated.

In order to verify the study hypotheses, Mplus 8 was used to test a Structural Equation Model (SEM); the method of estimation was Maximum Likelihood (ML). The model was assessed by several goodness-of-fit criteria [56] : the χ^2^ goodness-of-fit statistic; the Root Mean Square Error of Approximation (RMSEA); the Comparative Fit Index (CFI); the Tucker Lewis Index (TLI); and the Standardized Root Mean Square Residual (SRMR). Finally, the bootstrapping procedure was used to test the significance of the possible indirect effects [57]. To control for common method variance issues, we conducted Harman’s single-factor test [58] through a confirmatory factor analysis (CFA; ML solution). CFA results indicated that one single factor could not account for the variance in the data [χ^2^(434) = 1945.60, *p* < 0.001, RMSEA = 0.12, CFI = 0.47, TLI = 0.43, SRMR = 0.11].

## Results

A total of 231 students returned the questionnaire fully completed between September 2021 and January 2022; 73.6% were female and 17.7% were male (8.7% chose not to provide information on gender). The mean age was 21.52 years (SD = 2.37).

Table 1 shows the means, standard deviations, and correlations between the study variables, as well as their internal consistencies. The results show that the GPA positively correlated with work engagement, academic fit, and self-efficacy; work engagement showed a negative correlation with exhaustion and a positive correlation with academic fit, internal locus of control, and self-efficacy; academic fit also showed a positive correlation with both internal locus of control and self-efficacy; finally, internal locus of control and self-efficacy positively correlated with each other.

**Table 1:** Means, standard deviations, correlations and Alpha coefficients of all study variables

	M	SD	1	2	3	4	5	6
1. GPA	26.18	1.87	-					
2. Engagement	4.38	1.00	0.25^**^	0.84				
3. Exhaustion	3.32	1.35	-0.12	-0.32^**^	0.86			
4. Academic fit	3.92	0.42	0.19^**^	0.54^**^	-0.30^**^	0.79		
5. Internal LoC	3.12	0.61	-0.05	0.21^**^	-0.22^**^	0.25^**^	0.73	
6. Self-efficacy	3.28	0.71	0.15^*^	0.35^**^	-0.24^**^	0.39^**^	0.30^**^	0.80

** *p* < 0.05; * *p* < 0 0.01; alpha coefficients on the diagonal

**Fig. 2:**
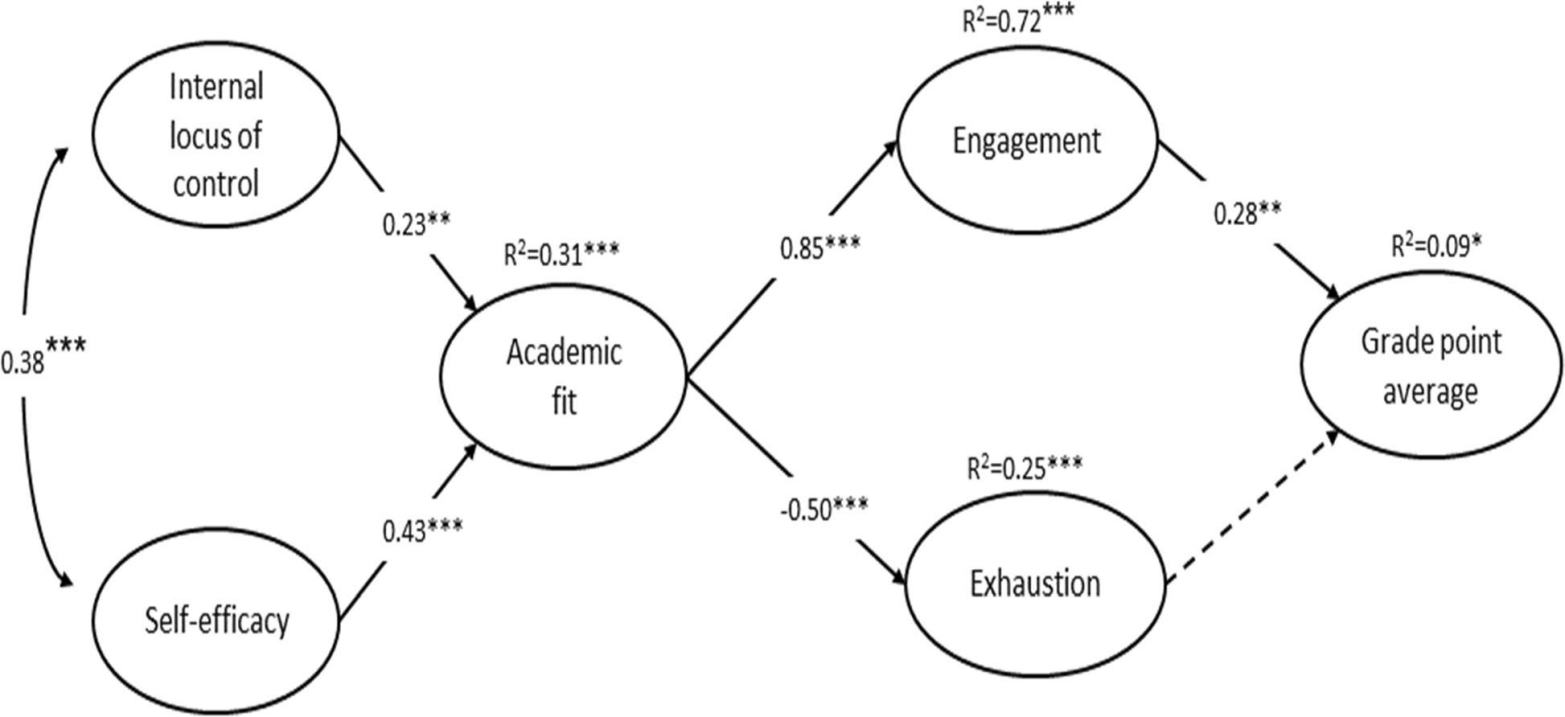
The final model (standardized path coefficients, *** *p* < 0.001; ** *p* < 0.01; * *p* < 0.05). Discontinuous line indicates a non-significant relationship

The study hypotheses were tested through a full-SEM that fitted to the data well: X^2^ (290) = 477.04, *p* < 0.001, CFI = 0.91, TLI = 0.90, RMSEA = 0.05 (0.04, 0.06), SRMR = 0.06. Standardized factor loadings ranged from 0.46 to 0.89. As shown in Fig. 2, both internal locus of control and self-efficacy had a positive relationship with academic fit, which was in turn positively related to work engagement and negatively related to exhaustion. Finally, only work engagement showed a positive relationship with GPA. The model explained about 31% of the variation in academic fit, 72% in work engagement, 25% in exhaustion, and 9% in GPA. Table 2 reports the results of the bootstrapping procedure, which confirmed that all the indirect effects were statistically significant.

**Table 2:** Indirect effects using bootstrapping (2,000 replications)

Indirect effects	Est.	S.E.	p	CI 95%
Loc -> AcFit -> Eng	0.20	0.07	0.005	0.06, 0.33
SEff -> AcFit -> Eng	0.37	0.07	< 0.001	0.23, 0.51
AcFit -> Eng -> GPA	0.24	0.07	0.001	0.10, 0.39
Loc -> AcFit -> Eng -> GPA	0.06	0.03	0.029	0.01, 0.11
SEff -> AcFit -> Eng -> GPA	0.10	0.04	0.004	0.04, 0.19
Loc -> AcFit -> Exh	-0.11	0.04	0.008	-0.21, -0.03
SEff -> AcFit -> Exh	-0.21	0.05	< 0.001	-0.32, -0.12

## Discussion

The goal of this research was to evaluate the linkages between psychological characteristics (internal locus of control and self-efficacy), academic fit, well-being (engagement and exhaustion), and veterinary student’s academic performance. Our findings contribute to veterinary education literature by providing evidence that the two psychological characteristics and academic fit are positively related with veterinary student’s well-being and academic performace.

As predicted in the first Hypothesis (H1), the findings confirmed a positive relationship between both internal locus of control and self-efficacy with academic fit. These findings are in line with those by Judge and Bono [59] who found a positive relationship between internal locus of control and self-efficacy with the perceived fit in the work environment. Our data suggest that students with an internal locus of control and higher levels of self-efficacy are more likely to fit in the academic environment. It can be expected that internally oriented students who believe that the events that occur in their environment are under their control, and who have high self-efficacy and believe in their ability to complete academic tasks successfully will fit more easily into the academic environment [6, 28]. In the present study we also found an indirect relationship between both internal locus of control and self-efficacy and academic performance mediated by academic fit and engagement. To explain this finding, we can suppose that locus of control and self-efficacy facilitate academic fit and engagement in the learning environment and allow students to achieve academic success. Previous studies have already revealed a positive relationship between both internal locus of control and self-efficacy with students’ academic performance [23, 60]; our study sheds light on the overall dynamic by demonstrating the role of important variables that mediate this relationship, namely academic fit and engagement. It seems reasonable that these students enjoy the learning experience, find the courses to match their interests, have their needs met by attaining their academic goals, and use their abilities and competencies in their courses. Since these students believe that they have the control and all the necessary capabilities to learn all the subjects pertaining to their degree course and to achieve their academic goals, they also experience a sense of satisfaction as they complete their education [6, 47, 61]. This can be reflected in an improved GPA.

The second finding of the present study is that academic fit is positively related to engagement and negatively related to exhaustion, thus confirming Hypothesis 2. To the best of our knowledge, this study is the first to demonstrate an effect of academic fit on engagement. One possible reason for this relationship could be that when students perceive fit with their courses and learning environment, they are more satisfied with their study activities and this leads to higher levels of engagement [62].

This study also revealed a significant negative relationship between veterinary students’ academic fit and exhaustion. This means that exhaustion in students can be predicted through academic fit adjustment. For example, if veterinary students are unable to fit to their studies, they will become incompatible with the learning environment, which can easily lead to academic exhaustion. Importantly, the effect of academic fit on students’ academic achievement was shown to be mediated through engagement. In other words, learners with high levels of academic fit showed higher engagement, and this can lead to higher academic achievement. According to Schmid (2008), the need to perceive fit in the academic environment is fundamental for the success of a student [5]. Thus, when a course meets a student’s interests and needs, we would expect them to make more effort and become more engaged in the learning process. As a consequence, they are more likely to achieve higher exam grades. This is a very relevant finding for veterinary medicine because it encourages veterinary schools to design and develop courses that match the student’s interests and needs, which will in turn directly impact upon their engagement, considered as a major factor for student success.

The final finding from this study was that engagement was positively related with academic performance, whereas exhaustion was not significantly associated with it, despite other studies have found this association [63, 64]. Thus, Hypothesis 3a was confirmed and Hypothesis 3b was rejected.

Consistent with the findings of the present study in regard to the positive relationship of engagement with academic performance, the results of Casuso-Holgado et al. and Gunuc showed that engagement was positively involved in academic achievement [42, 50]. In their meta-analysis study, Lei, Cui and Zhou also showed a positive correlation between engagement and academic achievement [51]. This implies that student engagement is one of the most important predictors of academic achievement. Indeed, veterinary educators need to do more to promote student engagement as it directly impacts the students’ academic performance.

## Conclusions

In conclusion, our structural model implies that psychological characteristics, academic fit and engagement influence the veterinary student’s academic performance. Our study revealed a positive relationship between both internal locus of control and self-efficacy with academic fit. Moreover, an indirect relationship was found between both internal locus of control and self-efficacy with the academic performance mediated by academic fit and engagement. In addition, academic fit resulted positively related with engagement and negatively related with exhaustion. Lastly, only student engagement demonstrated a positive direct relationship with academic performance. These findings have important implications for research and practice as described below.

### Limitations and future research

The main limitation of the study is that it has a cross-sectional design, which did not allow us to establish causal relationships between variables [65]. Future research should use longitudinal approaches to further explore the hypotheses and track the relationship between psychological dimensions, academic fit, student well-being, and academic performance over time. Another limitation is the use of self-reported data, which may have inflated the results due to the tendency of respondents to answer in a consistent manner. However, the study has the advantage of having used an objective indicator to measure academic performance. In the future, it would be interesting to consider other-reported measures (e.g., teacher or peer evaluation). In addition, contextual variables should also be considered in future studies in order to examine the role of, for example, teaching methods or academic programs in influencing student well-being and performance.

### Practical implications

Veterinary educators and curriculum planners should pay more attention to improving the academic fit of veterinary students by understanding their interests and needs better since it is the best predictor of students’ well-being. Studies have shown that well-being leads to a greater health in students as well as lower suicide rates. In particular, the general well-being of students was shown to be a strong protective factor for suicide prevention [66]. Because veterinary students are reported to be at higher risk of suicide compared with other student group [67], it is crucial that their well-being be promoted within the learning environment. Accordingly, the academic fit of veterinary students deserves to be taken into special consideration.

Results of our study also suggest that teachers in veterinary schools can reduce the veterinary students’ exhaustion through academic fit adjustment. Therefore, veterinary educators and curriculum planners should frequently seek for positive interventions in order to help veterinary students maintain a balance between learning activities and learning environment; i.e., the better the academic fit of veterinary students, the less likely they are going to become exhausted.

## Data Availability

The datasets generated and/or analysed during the current study are available from the corresponding author on reasonable request.

## Abbreviations

GPA: Grade point average
PE: Person environment
QR code: Quick response code
UWES-S: Utrecht work engagement scale for students
SPSS: Statistical package for social sciences
SEM: Structural equation model
ML: Maximum likelihood
RMSEA: Root mean square error of approximation
CFI: Comparative fit index
TLI: Tucker lewis index
SRMR: Standardized root mean square residual
CFA: Confirmatory factor analysis
